# Beyond accuracy: how often and in which direction diagnostic tests err

**DOI:** 10.11613/BM.2026.030101

**Published:** 2026-10-15

**Authors:** Farrokh Habibzadeh

**Affiliations:** Department of Medical Journalism, Shiraz University of Medical Sciences, Shiraz, Iran

**Keywords:** sensitivity, specificity, diagnostic errors, false positive reactions, false negative reactions, biostatistics

## Abstract

Diagnostic tests are commonly evaluated using sensitivity, specificity, predictive values, likelihood ratios, and receiver operating characteristic (ROC) curve analysis. Although essential, these measures do not fully answer two practical questions frequently raised in clinical medicine: how often a test makes mistakes, and whether those mistakes are predominantly false positive or false negative. This article introduces two complementary measures that address these questions: the number needed to misdiagnose (NNM), which expresses the frequency of diagnostic error in intuitive clinical terms, and the misdiagnosis direction index (MDI), which indicates whether errors are mainly false positive or false negative. A weighted MDI is also introduced for unequal clinical harms of errors. Together with conventional indices such as Youden’s index, these measures provide a broader framework for interpreting diagnostic performance.

## Graphical abstract

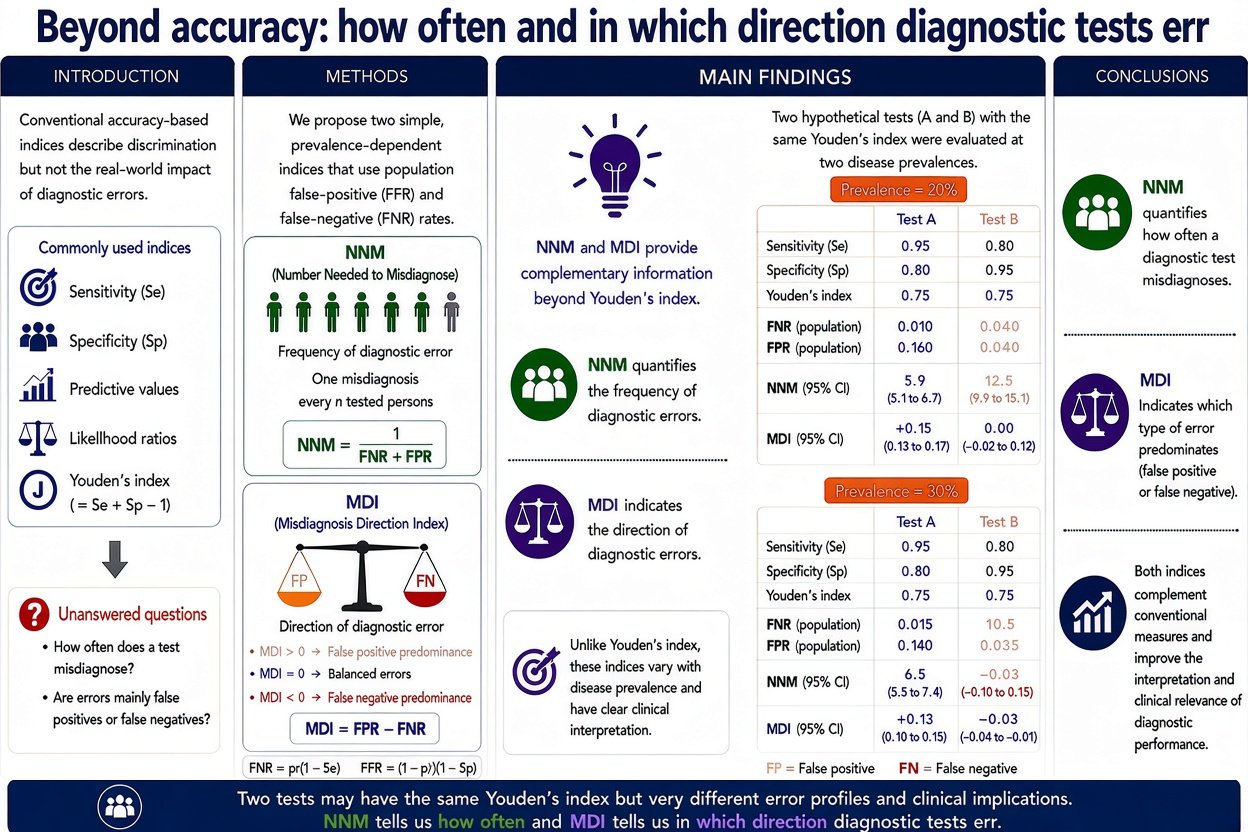

## Highlights

• Conventional accuracy metrics do not quantify error frequency or direction

• Number needed to misdiagnose (NNM) shows how often one misdiagnosis occurs

• Misdiagnosis direction index (MDI) shows whether errors are mainly false positive or false negative

• Weighted MDI incorporates unequal clinical consequences

• NNM and MDI complement conventional diagnostic indices

## Introduction

Diagnostic tests are integral to screening, diagnosis, prognosis, therapeutic monitoring, and clinical decision-making (*1*-*3*). Their results often determine whether further investigations are pursued, treatment is initiated, or disease is ruled out. Accordingly, understanding how test performance is measured and interpreted is fundamental to evidence-based medicine.

### Conventional performance measures and their limitations

The common performance indices used to evaluate diagnostic tests include sensitivity (Se), specificity (Sp), predictive values, likelihood ratios, and receiver operating characteristic (ROC) curve features (*4*, *5*). Each of these measures addresses a different clinical or methodological question (*1*, *5*-*8*). A detailed overview of these conventional indices has recently been published and will not be repeated here (*1*).

Briefly, Se estimates the probability that a diseased person has a positive test result; and Sp, the probability that a non-diseased person has a negative result (*6*). Positive and negative predictive values estimate the probability of presence or absence of a disease after the test result is known, and therefore depend strongly on disease prevalence (*1*). Likelihood ratios quantify how strongly a positive or negative result changes disease odds and are particularly useful in Bayesian interpretation of test results (*8*, *9*). Receiver operating characteristic curves summarize the trade-off between Se and Sp across different cut-off values; they are widely used for tests with continuous results (*5*, *7*).

Youden’s index (calculated as Se + Sp – 1) can be interpreted as the net probability of correct classification beyond chance, representing the difference between the probabilities of a positive test result in diseased and non-diseased individuals; the index ranges from 0 (useless test) to 1 (perfect test) (*10*). However, it does not indicate how many errors occur in a specific population because that depends on disease prevalence. Nor does it indicate whether errors are mainly false positive (FP) or false negative (FN). Therefore, Youden’s index, while useful for overall discrimination, shares similar limitations regarding error frequency and direction.

Although indispensable, the conventional performance measures do not directly answer two practical questions frequently raised by clinicians and policy-makers: 1) How often does this test make mistakes in the population where it is used?; 2) Are those mistakes mainly FP or FN?

These questions are paramount for clinicians because the consequences of the two error types may differ substantially. A FP result may lead to anxiety, unnecessary investigations, overtreatment, labeling, and increased healthcare costs. In contrast, a FN result may delay diagnosis, postpone treatment, worsen prognosis, or facilitate disease transmission.

Traditional indices address these concerns only partially. Sensitivity reflects the ability to minimize FN results, whereas Sp reflects the ability to minimize FP results; however, neither directly quantifies the overall population burden of diagnostic error (*6*). Similarly, Youden’s index summarizes discriminatory performance but does not indicate whether the remaining errors are predominantly FP or FN (*10*).

The number needed to misdiagnose (NNM) was proposed as a clinically intuitive summary of error frequency: the number of persons who need to be tested for one misdiagnosis (either FP or FN) to occur (*11*). While useful, NNM, however, does not indicate the direction of those errors.

This article therefore presents a practical framework that complements conventional diagnostic indices by focusing on two additional dimensions of test performance: the frequency of misdiagnosis and the direction of misdiagnostic error.

## How often does a test err? Number needed to misdiagnose

The population false-negative rate (FNR) and false-positive rate (FPR) are derived from the data presented in Table 1 using the following equations (Eqs):



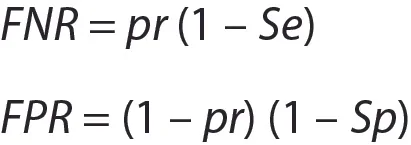



**Table 1:** Four possible outcomes when the results of a diagnostic test are compared with the results of a gold-standard (reference standard) test

		**Disease**		
		Present	Absent	
**Test Result**	Positive	TP n pr Se	FP n (1–pr) (1–Sp)	TP + FP
	Negative	FN n pr (1–Se)	TN n (1–pr) Sp	FN + TN
		TP + FN n pr	FP + TN n (1–pr)	n
In each cell, the expected frequencies in a population of n tested individuals, assuming disease prevalence, and test sensitivity and specificity. The expected number of true positives (TP) is n pr Se; false positives (FP), n (1 − pr)(1 − Sp); false negatives (FN), n pr (1 − Se); and true negatives (TN), n (1 – pr) Sp. These quantities form the basis for calculating the population false-negative rate (FNR), false-positive rate (FPR), number needed to misdiagnose (NNM), and misdiagnosis direction index (MDI). TP – true-positive. FP – false-positive. FN – false-negative. TN – true-negative. Se – sensitivity. Sp – specificity. pr – prevalence. n – total number of tested individuals.					

where pr represents prevalence; Se, sensitivity; and Sp, specificity. The NNM, by definition, is (*11*):



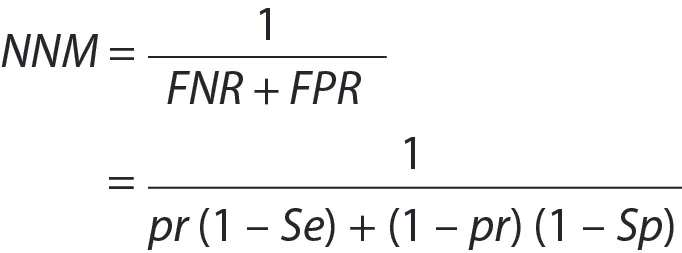



Number needed to misdiagnose is analogous to the number needed to treat in therapeutic studies. Rather than describing treatment benefit, it expresses diagnostic error in intuitive clinical units. The higher the NNM, the better the test performs (*1*, *11*).

## Example

If the overall population error rate (FNR  +  FPR) is 0.05:



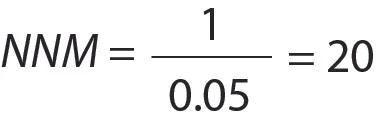



This means that, on average, one misdiagnosis occurs for every 20 tested individuals. Number needed to misdiagnose is clinically intuitive, but it does not indicate whether those errors are mainly FP or FN.

## In which direction does a test err? Addressing the limitation of NNM

To address the above-mentioned limitation of NNM, I define the misdiagnosis direction index (MDI) as follows:



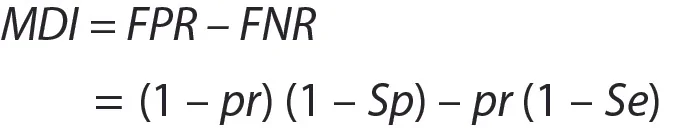



Misdiagnosis direction index measures the net population imbalance between FP and FN rates rather than the proportion of one error type among all misdiagnoses. Consequently, its magnitude reflects both the predominance of one error type and the overall burden of diagnostic error. The theoretical range of MDI is from −1 to +1. The absolute value reflects the degree of imbalance. A positive value shows FP predominance; a negative value, FN predominance; and zero, balanced error direction.

## Why the direction matters clinically

Two tests may have similar overall accuracy yet very different clinical implications. For example, in cancer screening, FNs may delay life-saving treatment; in infectious disease control, FNs may facilitate transmission; in low-risk population screening, FPs may cause more aggregate harm than FNs. Therefore, knowing the direction of diagnostic error may be as important as knowing its frequency.

## Weighted misdiagnosis direction index

Under certain circumstances, an FN result may be substantially more harmful than a FP result. Let C denote the relative harm of one FN compared with one FP, then:



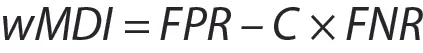



where wMDI represents the weighted MDI; a positive value indicates that the weighted FP burden predominates; a negative value, weighted FN burden predominates; and zero, weighted harms are balanced.

The value of C is not fixed and depends on the clinical context. It reflects the relative clinical, economic, or societal consequences of an FN result compared with a FP result. Selection of C should preferably be based on expert consensus, decision analysis, health-economic evaluation, or clinical guidelines (*7*, *12*).

## Example

Suppose a test has a population FPR of 0.04 and a population FNR of 0.01; then, the MDI is 0.03 (Eq. 3), reflecting FP dominance. Suppose that, in this example, a FN result is considered approximately five times more harmful than a FP result (C=5); then:



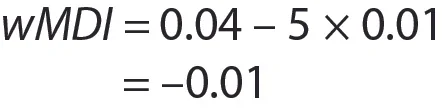



The wMDI is a weighted population error rate, expressed as a proportion of all tested individuals; it is not a relative risk or a percentage of misdiagnoses. After weighting, FNs become the dominant concern. Therefore, wMDI may help inform diagnostic threshold selection when the relative consequences of FP and FN results are unknown (*7*).

## Statistical uncertainty and confidence intervals

Like all estimates derived from sample data, NNM and MDI are subject to sampling variability. Therefore, point estimates should ideally be accompanied by confidence intervals (CIs).

## Confidence interval for NNM

Because NNM is a function of three estimated quantities, pr, Se, and Sp (Eq. 2), its sampling variability cannot be obtained directly from standard variance formulas. An approximate variance (Var) can be derived using the first-order Taylor series expansion (also termed ‘delta method’), which approximates a nonlinear function locally by a linear function in the neighborhood of the estimated values (*13*). This approximation expresses the variance of the derived measure in terms of the variances of its component estimates, weighted by the corresponding partial derivatives. From Eq. 2, the partial derivatives of NNM with respect to pr, Se, and Sp are:



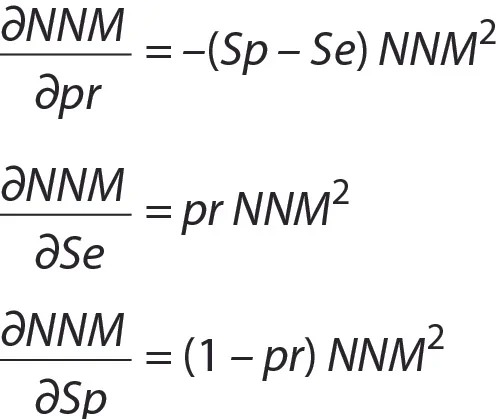



The approximate variance of NNM, using delta method, is (*13*):



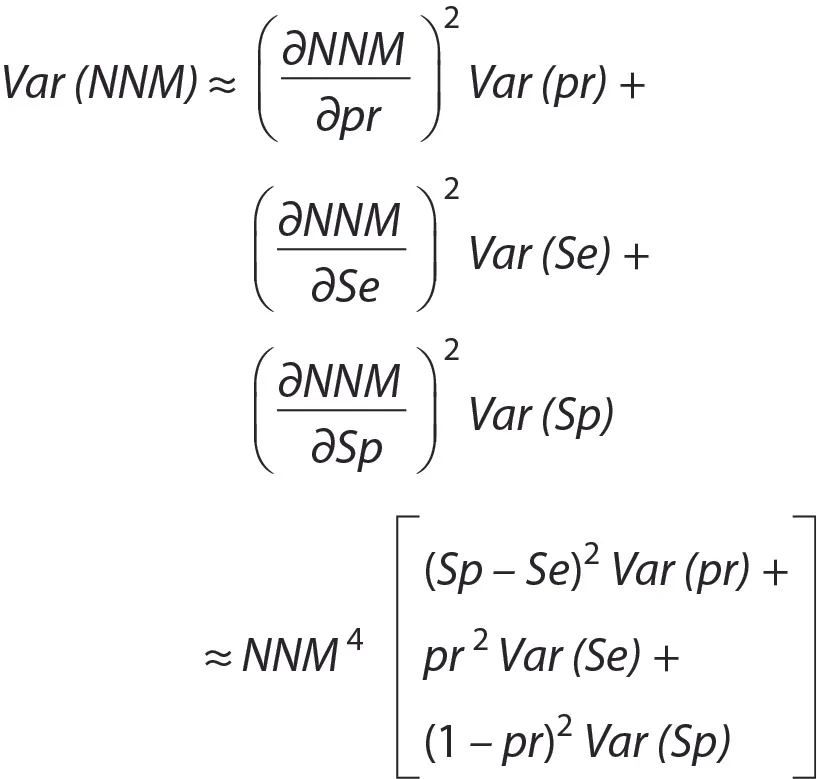



We have:



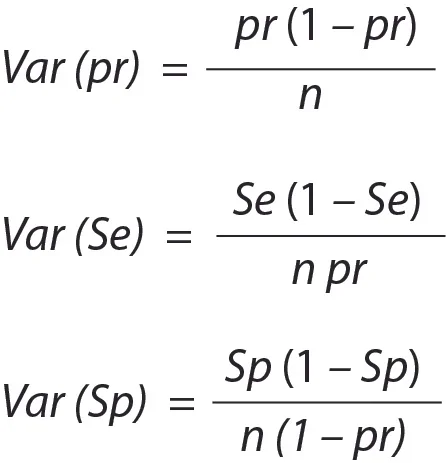



where n is the total number of tested individuals. Substituting the values into Eq. 7 and simplifying yield:



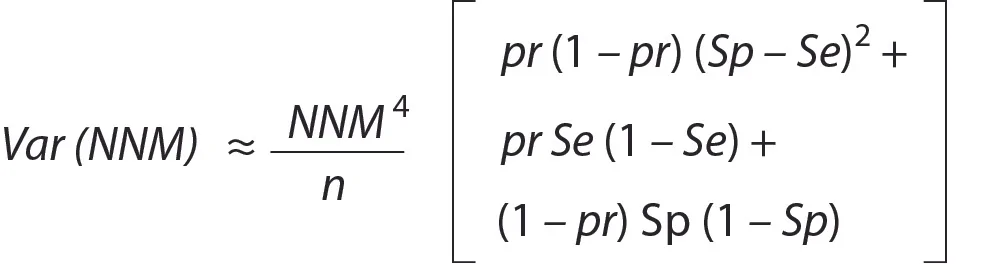



Because the variance of NNM is proportional to NNM^4^, variance increases dramatically as errors become rare (high NNM). Accordingly, an approximate 95% CI for NNM is:



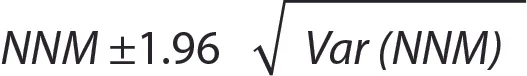



Because NNM is bounded below by zero and its variance increases rapidly as the misdiagnosis rate approaches zero, the resulting confidence interval may be asymmetric in practice; therefore, negative lower limits, if obtained, should be truncated at zero; in such cases, bootstrap-based intervals may provide more reliable estimates (*14*).

## Confidence interval for MDI

Misdiagnosis direction index is also a function of pr, Se, and Sp. From Eq. 3, the partial derivatives of MDI with respect to pr, Se, and Sp are:



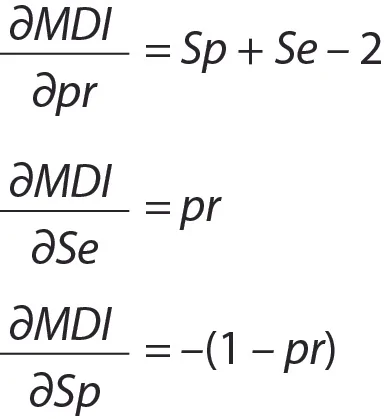



Using delta method, the approximate variance of MDI is:



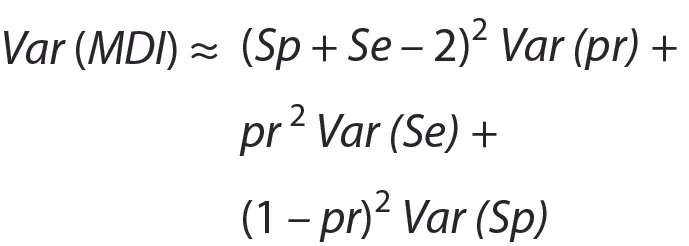



Substituting values from Eq. 7 in Eq. 11 and simplifying give:



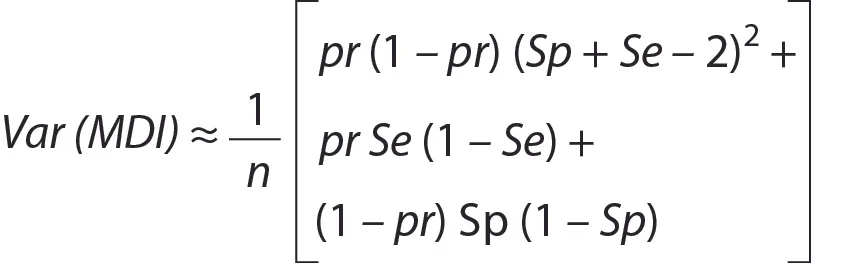



The 95% CI of MDI is therefore:



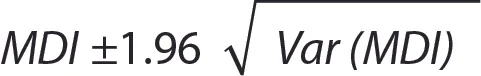



As before, when sample size is small or proportions are extreme, bootstrap confidence intervals may be preferable (*14*).

The following example is hypothetical and is intended solely to illustrate the proposed indices. Nevertheless, the situation reflects a common problem in laboratory medicine, where two competing assays or diagnostic strategies may exhibit similar overall discriminatory performance while differing substantially in the balance between Se and Sp. Such differences may have important clinical consequences that are not apparent from Youden’s index alone but are captured by the NNM and MDI.

## Worked example: complete calculation of NNM, MDI and their 95% CI

Consider two hypothetical diagnostic tests evaluated in a population of 1000 individuals with a disease prevalence of 20%. Thus, 200 individuals are diseased and 800 are disease-free.

The two tests were selected intentionally to demonstrate different error profiles. Suppose Test A has a Se of 0.95 and a Sp of 0.80, hence a Youden’s index of 0.75 (0.95 + 0.80 – 1). The expected outcome values of the test are presented in Table 2. The population FNR and FPR are then 0.01 and 0.16, respectively (Eq. 1). The total misdiagnosis rate is thus 0.17 (FNR + FPR), translating to an NNM of 5.9 (95% CI, 5.1 to 6.7) (Eqs. 2, 8 and 9). The corresponding MDI is 0.15 (0.13 to 0.17) (Eqs. 3, 12 and 13). On average, one misdiagnosis occurs for approximately every six tested individuals. The positive MDI and its 95% CI excluding zero indicate that diagnostic errors are predominantly FP.

**Table 2:** Expected classification table for Test A (sensitivity 0.95, specificity 0.80) in a hypothetical population of 1000 individuals with disease prevalence 20%

		**Disease**		
		Present	Absent	
**Test Result**	Positive	190	160	250
	Negative	10	640	650
		200	800	1000
Of the 1000 individuals, 200 are expected to have the disease and 800 are expected to be disease-free. Applying a sensitivity of 0.95 yields 190 true-positive and 10 false-negative results. Applying a specificity of 0.80 yields 640 true-negative and 160 false-positive results. The corresponding population false-negative rate is 0.01 (10/1000), the false-positive rate is 0.16 (160/1000), the total misdiagnosis rate is 0.17 (170/1000), and the number needed to misdiagnose is 5.9. This example illustrates a test with relatively frequent false-positive results.					

Now suppose another test, Test B, having a Se of 0.80 and a Sp of 0.95, hence a Youden’s index of 0.75 (0.80 + 0.95 – 1). Table 3 shows the classification table. Given the population FNR of 0.04, and a FPR of 0.04, the NNM is 12.5 (95% CI, 9.9 to 15.1); the MDI is 0 (-0.02, +0.02). This test produces one misdiagnosis for every 12.5 tested individuals, substantially outperforming Test A in overall error frequency. The MDI is zero, indicating no evidence of directional imbalance between FP and FN errors.

**Table 3:** Expected classification table for Test B (sensitivity 0.80, specificity 0.95) in a hypothetical population of 1000 individuals with disease prevalence 20%.

		**Disease**		
		Present	Absent	
**Test Result**	Positive	160	40	200
	Negative	40	760	800
		200	800	1000
Of the 1000 individuals, 200 are expected to have the disease and 800 are expected to be disease-free. Applying a sensitivity of 0.80 yields 160 true-positive and 40 false-negative results. Applying a specificity of 0.95 yields 760 true-negative and 40 false-positive results. The corresponding population false-negative rate is 0.04 (40/1000), the false-positive rate is 0.04 (40/1000), the total misdiagnosis rate is 0.08 (80/1000), and the number needed to misdiagnose is 12.5. This example illustrates a test with substantially fewer overall errors and a balanced distribution of false-positive and false-negative results.				

Both tests have identical Youden’s indices of 0.75 (Table 4), indicating similar overall discriminatory ability (*10*). However, NNM and MDI reveal clinically important differences in error frequency and direction. Test A misdiagnoses one in every six patients (predominantly FPs), while Test B misdiagnoses one in every 12.5 patients with balanced error distribution. This demonstrates that Youden’s index alone cannot capture the clinical utility differences between tests with similar discrimination but different error profiles.

**Table 4:** Summary of the diagnostic performance measures for two hypothetical diagnostic tests evaluated at disease prevalence of 20% and 30%

**Index**	**Prevalence 20%**		**Prevalence 30%**	
	**Test A**	**Test B**	**Test A**	**Test B**
Sensitivity	0.95	0.80	0.95	0.80
Specificity	0.80	0.95	0.80	0.95
False-negative rate	0.010	0.040	0.015	0.060
False-positive rate	0.160	0.040	0.140	0.035
Youden’s index	0.75	0.75	0.75	0.75
NNM (95% CI)	5.9 (5.1 to 6.7)	12.5 (9.9 to 15.1)	6.5 (5.5 to 7.4)	10.5 (8.5 to 12.5)
MDI (95% CI)	0.15 (0.13 to 0.17)	0.00 (-0.02 to 0.02)	0.13 (0.10 to 0.15)	-0.03 (-0.04 to -0.01)
Both tests have identical sensitivities, specificities, and Youden’s indices at each prevalence, indicating equivalent overall discriminatory performance. However, changing disease prevalence alters the population false-negative and false-positive rates and, consequently, the NNM and the MDI. The results illustrate that tests with identical Youden’s indices may differ substantially in the frequency and direction of diagnostic errors and that these differences vary with disease prevalence. NNM – number needed to misdiagnose. MDI – misdiagnosis direction index. CI – confidence interval.				

As a real-world laboratory example, consider measurement of prostate-specific antigen (PSA) for prostate cancer screening. Lowering the PSA decision threshold generally increases Se but reduces Sp, leading to more FP results and unnecessary prostate biopsies. Conversely, raising the threshold improves Sp but increases FN results, potentially delaying cancer diagnosis. Conventional measures such as Se, Sp, and Youden’s index summarize diagnostic discrimination, whereas the NNM additionally quantifies how frequently misdiagnoses (either FP or FN) occur in the screened population and the MDI indicates whether the remaining errors predominantly represent FP or FN classifications. These complementary measures may therefore facilitate selection of clinically appropriate PSA decision thresholds. Furthermore, because the clinical consequences of missing an aggressive prostate cancer differ from those of performing an unnecessary biopsy, different healthcare systems or clinical guidelines may reasonably assign different relative weights to FN and FP results when using the wMDI (Eq. 4).

## Comparison of two diagnostic tests

Although both tests would generally be considered diagnostically acceptable, they differ substantially in their clinical implications. Test A has higher Se and therefore minimizes FN results (more appropriate for ruling out a disease), but this occurs at the expense of frequent FP results (*1*, *6*, *15*). Its positive MDI indicates a strong tendency toward over-diagnosis. Test B, on the other hand, has lower Se but substantially higher Sp (more appropriate for ruling in a disease), resulting in fewer overall errors and balanced misclassification (Table 4).

This example illustrates how NNM and MDI together provide clinically relevant information not conveyed by conventional test performance measures alone. Number needed to misdiagnose answers how often does the test make mistakes; MDI answers in which direction are those mistakes occurring.

To further illustrate the influence of disease prevalence, Table 4 compares the two tests when the prevalence increases from 20% to 30%, while Se and Sp remain unchanged. Although both tests continue to have identical Youden’s indices of 0.75, their NNM and MDI values change appreciably. For Test A, the NNM increases modestly because the reduction in FP results outweighs the increase in FN results, while the MDI remains positive, indicating continued predominance of FP errors. In contrast, Test B shows a decrease in NNM and a shift of the MDI from approximately zero to a negative value, indicating that FN errors become more frequent than FP errors as disease prevalence increases. These findings demonstrate that, unlike Youden’s index, NNM and MDI incorporate the effect of disease prevalence and provide clinically relevant information regarding both the frequency and direction of diagnostic errors.

Number needed to misdiagnose and MDI vary as disease prevalence changes for tests with different Se and Sp profiles. Number needed to misdiagnose is strongly prevalence-dependent (Figure 1). Even when Se and Sp remain fixed, overall diagnostic performance may vary substantially across populations with different disease prevalence. Tests optimized for high Se often exhibit reduced NNM at low prevalence because FP results dominate (Figure 1).

**Figure 1:**
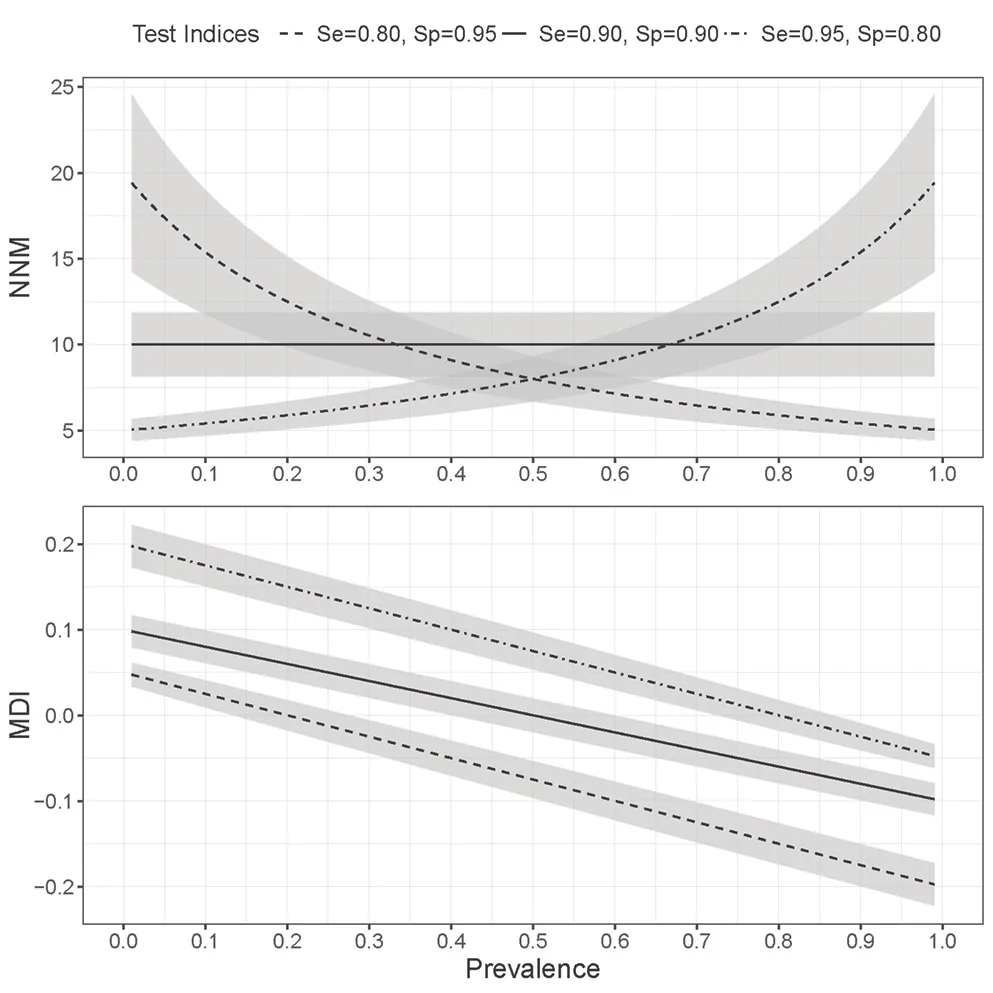
Effect of disease prevalence on the number needed to misdiagnose (NNM) and misdiagnosis direction index (MDI) for three tests with different sensitivity (Se) and specificity (Sp) profiles in 1000 tested individuals. Upper panel: NNM. Lower panel: MDI. Shaded areas represent approximate 95% confidence intervals (delta method). Note strong prevalence dependence of NNM and potential sign change in MDI.

Misdiagnosis direction index may change sign as prevalence changes. At low prevalence, FP errors often predominate, resulting in positive MDI values. As prevalence increases, FN errors become increasingly influential, potentially shifting MDI below zero (Figure 1, lower panel). The prevalence at which MDI crosses zero represents the equilibrium point where FP and FN misclassifications are equally frequent. This behavior highlights the importance of interpreting diagnostic performance in the clinical population of intended use.

For a given test under certain circumstances (constant Se, Sp, and disease prevalence), both MDI and NNM 95% CI widths decrease approximately in proportion to 1/√n as sample size increases (Figure 2). However, because *Var (NNM)* µ *NNM*^4^ (Eq. 8), tests with very low error rates (large NNM values) require considerably larger sample sizes to achieve precise estimates than tests with moderate NNM values. When comparing across different tests, those with larger NNM will show wider confidence intervals for the same sample size. Consequently, even modest uncertainty in Se, Sp, or prevalence may translate into substantial uncertainty in NNM when diagnostic errors are rare. This property emphasizes the importance of reporting confidence intervals alongside point estimates, particularly when very large NNM values are observed.

**Figure 2:**
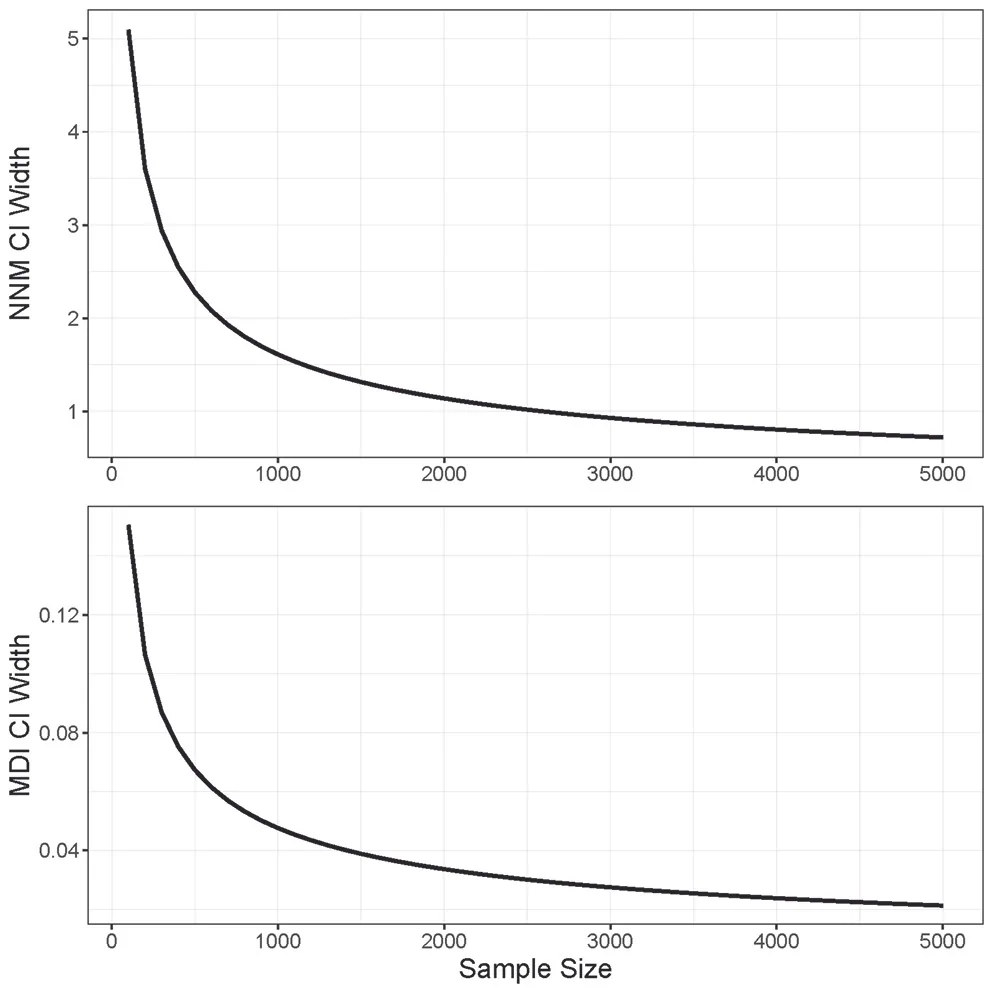
For the test (sensitivity of 0.95 and specificity of 0.80) and disease prevalence of 20%, the 95% confidence interval (CI) widths of both number needed to misdiagnose (NNM) and the misdiagnosis direction index (MDI) decrease as sample size (n) increases. Both follow approximately 1/√n relationships (note the different Y-axis scales). Both curves follow the same 1/√n decay because, at fixed prevalence, sensitivity, and specificity, the variances of NNM and MDI are each a constant divided by n; the distinctive property of NNM – its variance is proportional to NNM^4^ – becomes apparent only when comparing tests with different NNM values (Figure 1), not when varying sample size for a single test.

## Relationship with ROC curves and cut-off values

Changing the cut-off value of a quantitative test usually changes Se and Sp in opposite directions (*5*-*7*). Consequently, NNM and MDI also change. A lower cut-off often increases Se and reduces FNs but may increase FPs. A higher cut-off often increases Sp and reduces FPs, but may increase FNs (*5*, *15*). Therefore, cut-off selection should ideally consider not only ROC characteristics, but also the expected frequency and direction of errors in the intended population (*3*, *7*, *15*).

## Practical recommendations for reporting

When presenting diagnostic studies, authors should report Se, Sp, predictive values, likelihood ratios, and area under the ROC curve (AUC) as core measures. Where the clinical population prevalence is known or estimable, reporting NNM and MDI with their 95% CIs is also recommended. When the clinical consequences of FP and FN errors differ substantially, the wMDI should be reported alongside the clinical consequence ratio used.

## Conclusion

No single metric captures all aspects of diagnostic performance. Conventional indices such as Se, Sp, likelihood ratios, and ROC analysis remain essential, but they do not directly quantify how often a test errs or whether those errors are predominantly FP or FN. The NNM provides an intuitive measure of error frequency, while the MDI indicates the direction of diagnostic error. Together with established measures, these indices may provide a broader and more clinically meaningful understanding of test performance.

## Data Availability

All data generated and analyzed in the presented study are included in this published article.

## References

[1] Habibzadeh F. Diagnostic tests performance indices: an overview. Biochem Med (Zagreb). 2025;35:010101. 10.11613/BM.2025.010101 39974192 PMC11838712

[2] Habibzadeh P Yadollahie M Habibzadeh F. What is a “Diagnostic Test Reference Range” Good for? Eur Urol. 2017;72:859–60. 10.1016/j.eururo.2017.05.024 28558944

[3] Sox HC, Higgins MC, Owens DK, Schmidler GS. Medical decision making. Hoboken: John Wiley & Sons; 2024. 10.1002/9781119627876

[4] Florkowski CM. Sensitivity, specificity, receiver-operating characteristic (ROC) curves and likelihood ratios: communicating the performance of diagnostic tests. Clin Biochem Rev. 2008;29 Suppl 1:S83–7. 18852864 PMC2556590

[5] Altman DG Bland JM. Diagnostic tests 3: receiver operating characteristic plots. BMJ. 1994;309(6948):188. 10.1136/bmj.309.6948.188 8044101 PMC2540706

[6] Altman DG Bland JM. Diagnostic tests. 1: Sensitivity and specificity. BMJ. 1994;308(6943):1552. 10.1136/bmj.308.6943.1552 8019315 PMC2540489

[7] Habibzadeh F Habibzadeh P Yadollahie M. On determining the most appropriate test cut-off value: the case of tests with continuous results. Biochem Med (Zagreb). 2016;26:297–307. 10.11613/BM.2016.034 27812299 PMC5082211

[8] Habibzadeh F Habibzadeh P. The likelihood ratio and its graphical representation. Biochem Med (Zagreb). 2019;29:020101. 10.11613/BM.2019.020101 31015780 PMC6457916

[9] Goligher EC Heath A Harhay MO. Bayesian statistics for clinical research. Lancet. 2024;404:1067–76. 10.1016/S0140-6736(24)01295-9 39277290 PMC12051211

[10] Youden WJ. Index for rating diagnostic tests. Cancer. 1950;3:32–5. 10.1002/1097-0142(1950)3:1<32::AID-CNCR2820030106>3.0.CO;2-3 15405679

[11] Habibzadeh F Yadollahie M. Number Needed to Misdiagnose. Epidemiology. 2013;24:170. 10.1097/EDE.0b013e31827825f2 23232619

[12] Vickers AJ Elkin EB. Decision Curve Analysis: A Novel Method for Evaluating Prediction Models. Med Decis Making. 2006;26:565–74. 10.1177/0272989X06295361 17099194 PMC2577036

[13] Cox C. Delta method. In: Armitage P, Colton T, editors. Encyclopedia of Biostatistics. 2nd ed. Chichester: John Wiley & Sons; 2005.

[14] Efron B, Tibshirani RJ. An Introduction to the Bootstrap. New York: Chapman & Hall; 1993. 10.1007/978-1-4899-4541-9

[15] Newman TB, Kohn MA. Evidence-Based Diagnosis. New York: Cambridge University Press; 2009. 10.1017/CBO9780511759512

